# Do ancient wheats contain less gluten than modern bread wheat, in favour of better health?

**DOI:** 10.1111/nbu.12551

**Published:** 2022-05-13

**Authors:** Fred Brouns, Sabrina Geisslitz, Carlos Guzman, Tatsuya M. Ikeda, Ahmad Arzani, Giovanni Latella, Senay Simsek, Mariastella Colomba, Armando Gregorini, Victor Zevallos, Valerie Lullien‐Pellerin, Daisy Jonkers, Peter R. Shewry

**Affiliations:** ^1^ NUTRIM‐School for Nutrition and Translational Research in Metabolism Maastricht University Maastricht The Netherlands; ^2^ Institute of Applied Biosciences Karlsruhe Institute of Technology (KIT) Karlsruhe Germany; ^3^ ETSIAM Edificio Gregor Mendel Universidad de Córdoba Córdoba Spain; ^4^ Western Region Agricultural Research Centre National Agriculture and Food Research Organization (NAR0) Hiroshima Japan; ^5^ 48456 College of Agriculture Isfahan University of Technology Isfahan Iran; ^6^ Gastroenterology, Hepatology and Nutrition Division University of L’Aquila L’Aquila Italy; ^7^ 311308 Purdue University West Lafayette Indiana USA; ^8^ University of Urbino “Carlo Bo” Urbino Italy; ^9^ University of Northumbria Newcastle Upon Tyne UK; ^10^ IATE, INRAE Institut Agro Univ. Montpellier Montpellier France; ^11^ NUTRIM School for Nutrition and Translational Research in Metabolism Maastricht University Maastricht The Netherlands; ^12^ Rothamsted Research Harpenden Hertfordshire UK

**Keywords:** ancient grains, coeliac disease, FODMAP, gluten, gluten sensitivity, wheat

## Abstract

Popular media messaging has led to increased public perception that gluten‐containing foods are bad for health. In parallel, ‘ancient grains’ have been promoted with claims that they contain less gluten. There appears to be no clear definition of ‘ancient grains’ but the term usually includes einkorn, emmer, spelt and Khorasan wheat. Gluten is present in all wheat grains and all can induce coeliac disease (CD) in genetically susceptible individuals. Analyses of ‘ancient’ and ‘modern’ wheats show that the protein content of modern bread wheat (*Triticum aestivum*) has decreased over time while the starch content increased. In addition, it was shown that, compared to bread wheat, ancient wheats contain more protein and gluten and greater contents of many CD‐active epitopes. Consequently, no single wheat type can be recommended as better for reducing the risks of or mitigating the severity of CD. An estimated 10% of the population of Western countries suffers from gastrointestinal symptoms that lack a clear organic cause and is often referred to as irritable bowel syndrome (IBS). Many of these patients consider themselves gluten sensitive, but in most cases this is not confirmed when tested in a medical setting. Instead, it may be caused by gas formation due to fermentation of fructans present in wheat or, in some patients, effects of non‐gluten proteins. A significant overlap of symptoms with those of CD, IBS and inflammatory bowel disease makes a medical diagnosis a priority. This critical narrative review examines the suggestion that ‘ancient’ wheat types are preferred for health and better tolerance.

## INTRODUCTION

Over recent years, popular books and social media postings have suggested that the consumption of gluten in products made from modern types of bread and durum (pasta) wheats results in a range of adverse effects and contributes to chronic diseases including obesity. In addition, it has been suggested that modern varieties of bread wheat (*Triticum aestivum*), which is sometimes referred to as ‘common wheat’, have higher gluten contents compared to so‐called ‘ancient wheats’. It has also been claimed that the gluten present in modern wheat is poorly digested, leading to the presence of partially digested protein fragments in the small intestine that play a role in the intestinal pathology of coeliac disease (CD) and are linked to neurodegenerative conditions (gluten ataxia). In addition, it has been suggested that gluten is linked to the exacerbation of chronic brain disorders such as schizophrenia, depression, Alzheimer's disease and autism (Davis, 2012; Perlmutter, 2014). Although the latter claims have been shown to be unjustified based on absence of solid evidence (for example, see (Miller‐Jones, 2012)), intense activity in the social and popular media has led to the widely held public perception that gluten‐containing foods should be avoided by all. We therefore critically examine the suggestion that ‘ancient’ types of wheat contain less gluten and fewer CD‐active peptides (peptides containing epitopes – specific domains of sequences of amino acids that are recognised by the immune system) than modern bread wheat and are therefore a healthier option. We will do this by comparing claims and suggestions made in social media with the current scientific evidence base.

## WHAT ARE ‘ANCIENT’ GRAINS?

Wikipedia (Wikipedia, 2021), which is often consulted by individuals who have no access to scientific libraries, states that ‘ancient grains is a marketing term used to describe a category of grains and pseudo‐cereals that are purported to have been minimally changed by selective breeding over recent millennia, as opposed to more widespread cereals such as corn, rice and modern varieties of wheat, which are the product of thousands of years of selective breeding’. This description clearly lacks precision and there is no universally accepted definition of the term ‘ancient’ and ‘modern’ grains when applied to wheat.

The terms are sometimes applied to modern and older types of bread and durum wheat (also known as ‘pasta wheat’), defining modern as the most recently bred and widely cultivated and ‘ancient’ as older types that are no longer widely cultivated (having been superseded in terms of yield, agronomic performance and quality). These older types of bread and durum wheats, which may date back to more than a century, may correspond to unselected land races or the products of early breeding and should therefore be considered as ‘heritage’ or ‘heirloom’ wheats.

The term ‘ancient grains’ is more widely applied, and therefore used here to describe types of wheat which are suggested to correspond to types which were grown in antiquity but are only currently grown in small volumes today: einkorn, emmer, spelt and Khorasan wheat. To understand the relationships between these and modern types of bread and durum wheats it is necessary to briefly discuss the origin and evolution of wheat.

**FIGURE 1:**
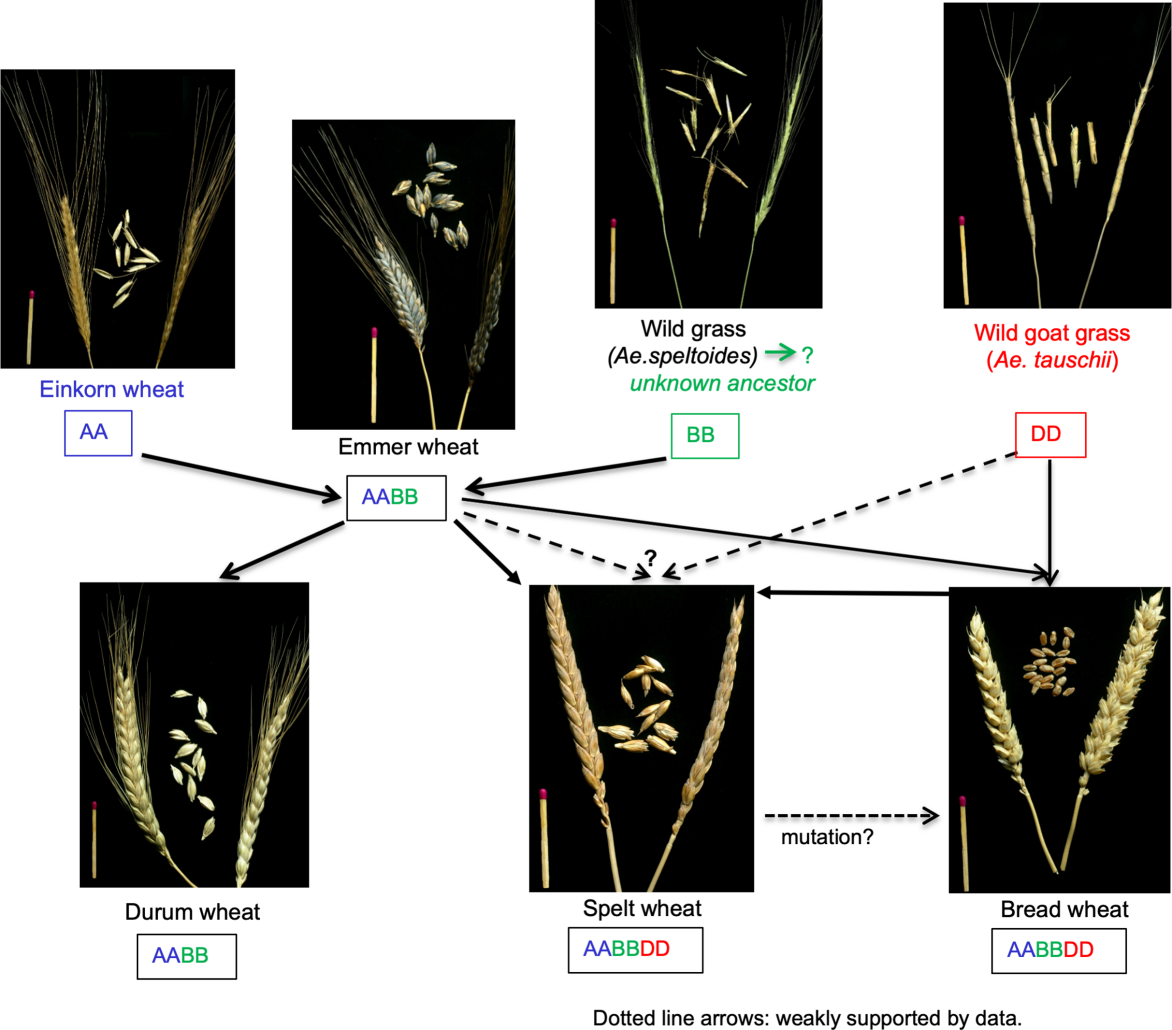
Condensed history of wheat. Natural crossing of early diploid wheat ancestors (with the AA and BB genomes) led to the development of tetraploid emmer wheat (AABB genomes), which subsequently became domesticated and diversified into durum wheat. Cultivated bread wheat (AABBDD genomes) arose about 10 000 BC, probably from the crossing of the tetraploid (AABB) emmer with diploid wild goat grass (DD). Recent genetic studies indicate that spelt wheat probably arose from the crossing of bread wheat (AABBDD) with emmer wheat (AABB). Overall, more than a dozen wheat subspecies exists and within each subspecies, thousands of different varieties are available in cultivation and/or gene banks. Furthermore, within each subspecies these varieties vary widely in their age (including land races and old and modern varieties), agronomic properties, and grain composition and food processing quality. Thus, many ‘older’ and more ‘recent’ varieties of bread wheat, spelt, durum and emmer exist. (Arzani & Ashraf, 2017; Dvorak et al., 2012; Faris, 2014; Feuillet et al., 2008; Honegger & Mertz, 2021; Matsuoka, 2011; Pont et al., 2019)

In simple terms, the history of wheat (as far as we currently know) extends some 500 000 years (Chalupska et al., 2008) (Figure 1). The earliest known form is the diploid species einkorn (two sets of chromosomes, AA), which exists in wild and cultivated forms. Natural crossbreeding of einkorn with a related wild grass (probably an unknown form of modern *Aegilops speltoides*) gave rise to a single tetraploid (four sets of chromosomes, AABB) wheat species which exists in three major forms (wild emmer, cultivated emmer and modern durum wheat) and minor forms (notably Khorasan wheat). Finally, the crossing of wild emmer with a different related grass species (goat grass, *Aegilops tauschii* also called *Triticum tauschii* and *Aegilops squarrosa*) gave rise to a single hexaploid (six sets of chromosomes, AABBDD) species with two main forms: spelt‐like hulled wheat and bread wheat.

The types of einkorn and emmer and also Khorasan wheat (*Triiticum turgidum* ssp. *turanicum*, a tretraploid wheat which includes the form known as Kamut^TM^) grown today appear to be descended from wheat types grown during the earliest cultivation, although both have undoubtedly been subjected to selection during their period of cultivation. Hence, both may be correctly termed ‘ancient’. This does not apply to spelt as the forms that are currently grown appear to have been derived from more recent hybridisations between hexaploid bread wheat and wild emmer, rather than from ancient spelt‐like hulled wheat, which may be extinct. Furthermore, modern commercial types of spelt have been subjected to plant breeding in the same way as modern bread and pasta wheats.

Genetic differences within and between species and types of wheat have an impact on grain composition and quality parameters for food processing which may affect consumer preferences. Some of these differences, for example, the content and composition of gluten, have also been suggested to have an impact on health and adverse reactions that may occur in susceptible individuals. This will be discussed in more detail below.

## DO ANCIENT WHEATS CONTAIN LESS GLUTEN THAN BREAD WHEAT?

**FIGURE 2:**
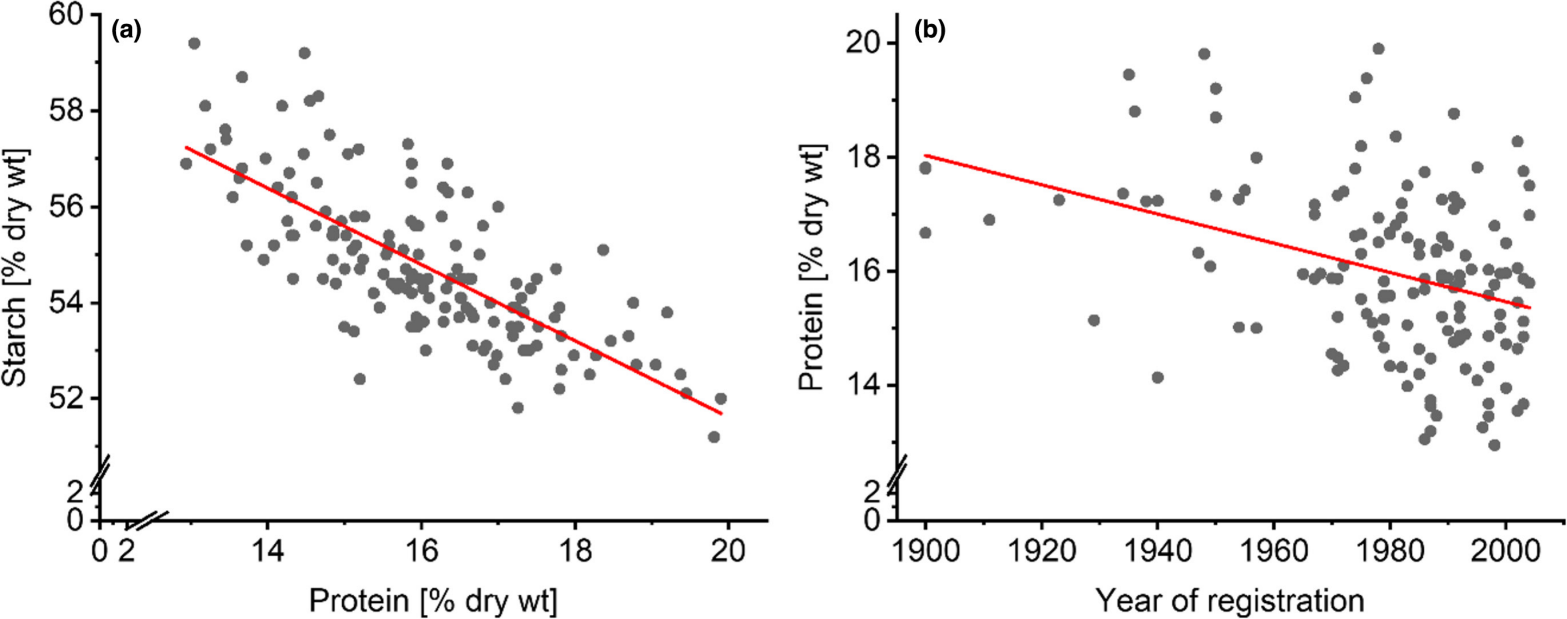
Analysis of 150 wheat lines (130 winter wheats and 20 spring wheats), all grown under identical circumstances in Martonvasar in Hungary showed that: (a) over time an increased starch content ‘dilutes’ the protein content in a linear fashion, resulting in (b) a decline in protein content over time. With courtesy of Shewry et al. (2020)

An extensive compositional analysis (Shewry et al., 2020) of 150 varieties of wheat obtained from seed banks (including older varieties from the 19th and early 20th centuries and modern types produced by intensive breeding) that were grown, harvested and milled under identical conditions, showed that the protein content of wheat has declined slightly from older to modern types (Figure 2). This decrease is associated with a parallel increase in the content of starch, which is responsible for the higher yields.

**FIGURE 3:**
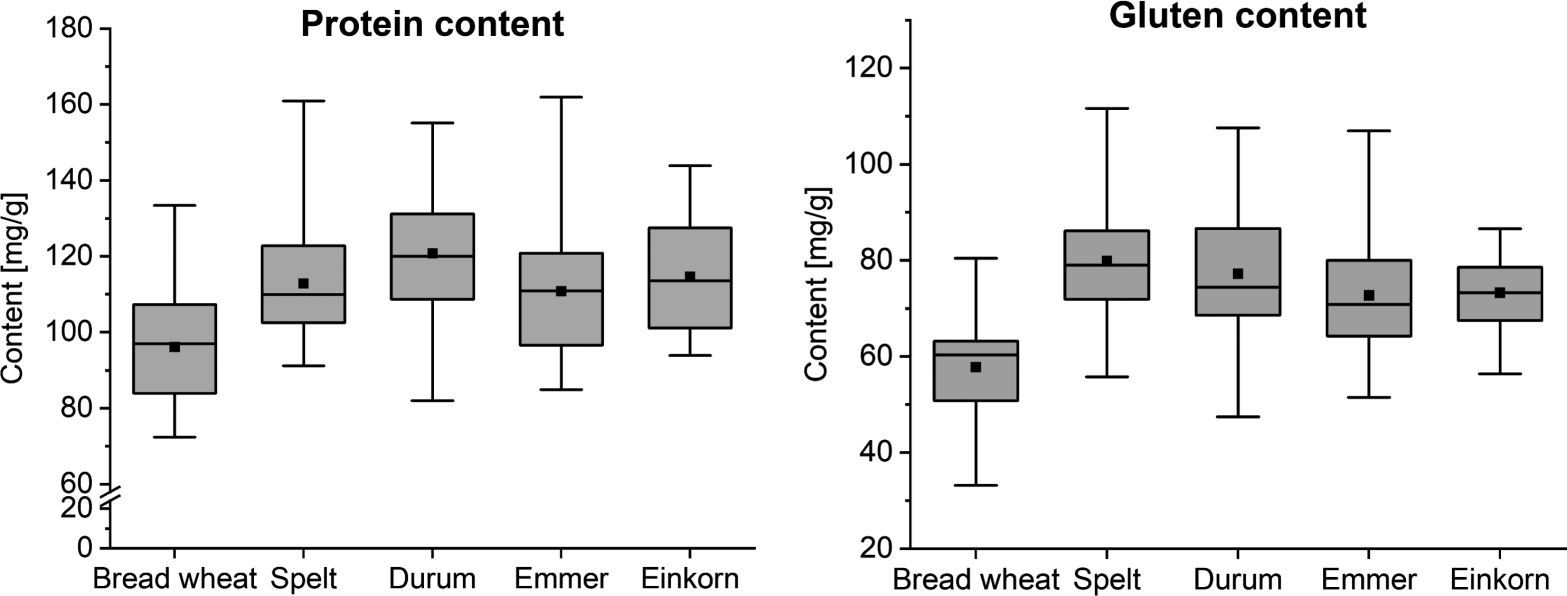
Ancient wheats contain more gluten. Total protein (left) and gluten content in bread wheat, spelt, durum wheat, emmer and einkorn (*n* = 15 cultivars grown at four locations (*n* = 60) in Germany). Modified with courtesy from Geisslitz et al. (2019)

Gluten is a mixture of storage proteins, contributing about 70%–80% of the total grain protein content. Hence, it is likely that if total protein decreases over time (Shewry et al., 2020), the gluten protein content will also decrease. Indeed, recent analysis (Geisslitz et al., 2019) showed this to be the case with spelt, emmer and einkorn having higher contents of total protein and gluten compared to modern bread wheats, but not higher contents than modern durum wheats (see Figure 3).

## DO ANCIENT WHEATS CONTAIN LESS GLIADIN, RESULTING IN FEWER TOXIC PEPTIDES?

Scientific studies have attempted to determine whether the composition and type of protein fragments (peptides) that remain undigested in our intestine (and therefore may induce inflammatory and immune responses) differ between modern bread wheat and ancient wheats. This question is relevant because these peptides are known to pose a risk for the development of coeliac disease (CD) in individuals who are genetically predisposed (this is the case in the part of the population [5%–40%] who express the haplotypes HLA‐DQ2 or DQ8). The proportion of susceptible individuals differs in different regions, as reviewed by (Brouns et al., 2019; Lebwohl et al., 2015) and it is estimated that only about 4% of these individuals actually develop CD, resulting in a mean prevalence of about 1% of the total population. This small percentage of genetically pre‐disposed individuals that actually do develop CD means that additional factors (other than gluten) must play a role in the initiation of the disease development.

**FIGURE 4:**
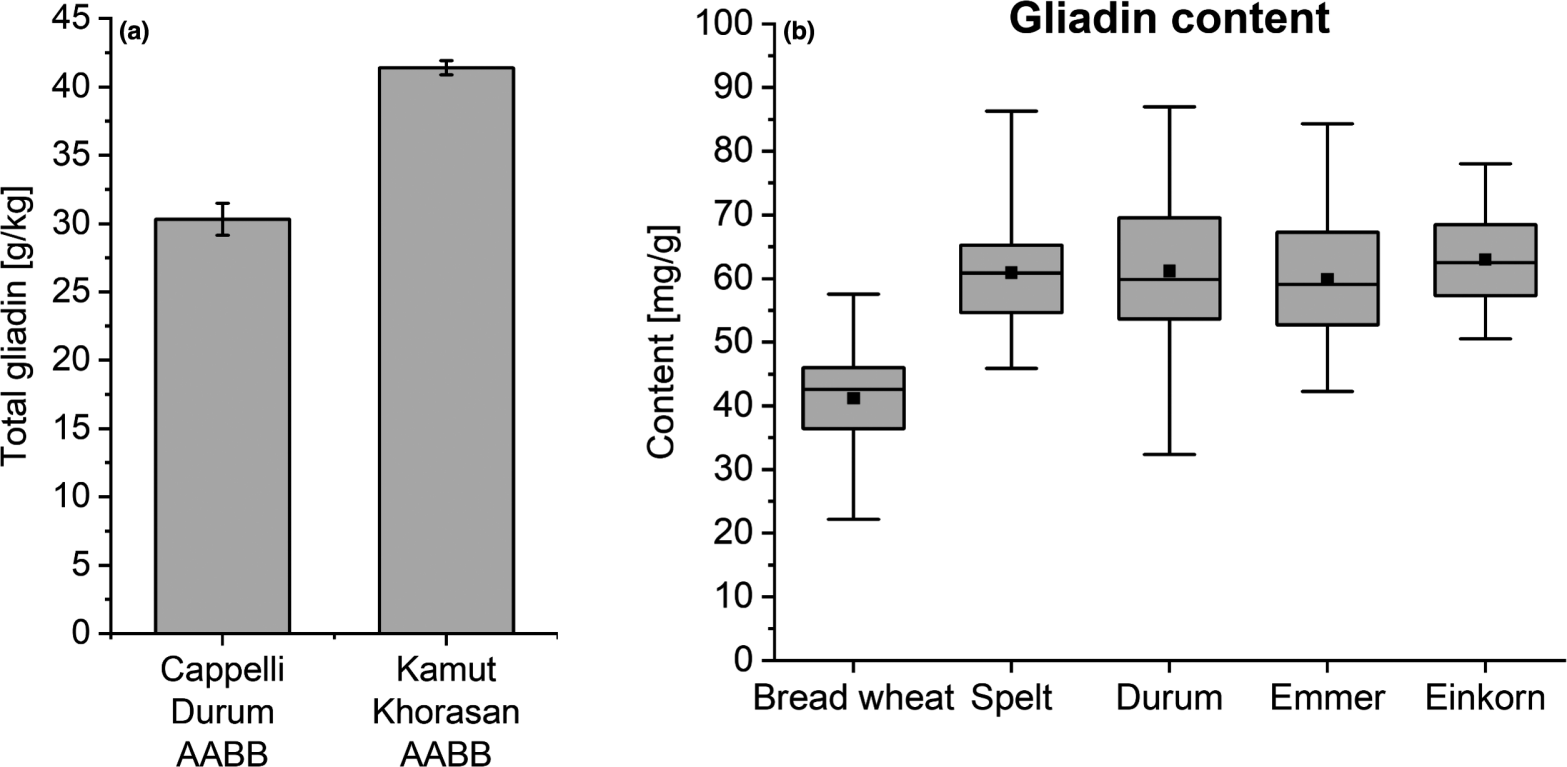
(a) Total gliadin contents of an ‘ancient’ wheat (Khorasan) and a traditional Italian heritage durum wheat (Senatore Cappelli). Figure kindly supplied by A. Gregorini, based on Gregorini et al. (2009). (b) Gliadin content in ‘ancient’ wheats (spelt, emmer and einkorn) compared to modern bread and durum wheat. With courtesy from Geisslitz et al. (2019)

**FIGURE 5:**
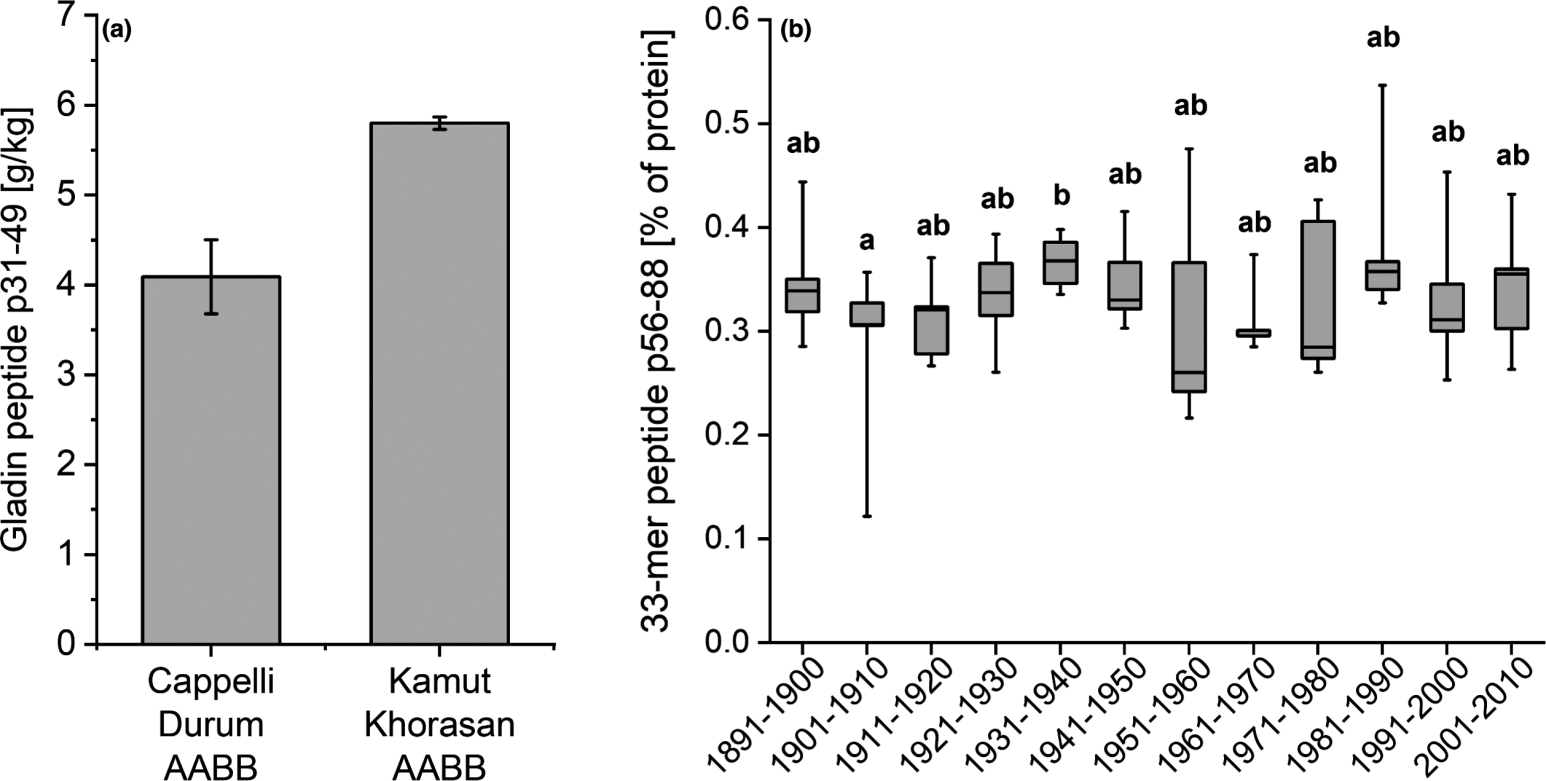
(a) Example of a selected gliadin derived peptide (so‐called p. 31–49), which was higher in ‘ancient’ wheat (Khorasan), compared to modern durum wheat (cultivar Cappelli). Figure kindly supplied by A. Gregorini, based on Gregorini et al. (2009). (b) Proportion of 33‐mer peptide based on protein content. Five wheat cultivars per decade of three subsequent harvest years were analysed. Modified from Pronin et al. (2021)

Gluten proteins comprise two fractions: gliadin and glutenin. Whereas glutenin is particularly important for determining dough elasticity and breadmaking quality, gliadin is the more important source of fragments resulting from protein digestion (peptides) that may cause adverse reactions in the gut. These fragments may contain core amino acid sequences which are able to trigger immune reactivity and the onset of CD in genetically susceptible individuals. These specific sequences to which antibodies bind are referred to as CD‐active epitopes. Although preliminary research suggested that wheat breeding had resulted in a higher content of some CD‐active peptides (van den Broeck et al., 2010), more recent work has shown that there is wide variation in the number and distribution of these epitopes within and between different types and varieties of wheat, with no clear differences between modern cultivars and older landraces which were not subjected to scientific breeding (Pronin et al., 2020; Ribeiro et al., 2016). By contrast, other studies have shown that the abundances of gliadins and many CD‐active epitopes present in gliadins are higher in ancient wheats compared to modern wheat (Colomba & Gregorini, 2012; Geisslitz et al., 2019; Gregorini et al., 2009; Prandi et al., 2017) (see Figures 4 and 5).

In a recent study (Pronin et al., 2020), 60 German winter wheat cultivars, registered between 1891 and 2010, were grown under controlled conditions for 3 years and analysed for protein content and gluten composition. It was concluded that the breeding of bread wheat from 1891 to 2010 contributed to increasing glutenin contents but decreasing total protein and gliadin contents, mainly of α‐ and γ‐gliadins, with no changes in the contents of soluble (albumin and globulin) proteins or of total gluten. Of particular interest is the content of the 33‐mer peptide (LQLQPFPQPQLPYPQPQLPYPQPQLPYPQPQPF; α2‐gliadin p. 56–88), which is a potent stimulator of ‘CD responses’ in vitro (Qiao et al., 2004). This peptide is only present in α‐gliadins encoded by the D‐genome (see Figure 1) and hence is lacking in the grains of einkorn, emmer and durum wheat, which all lack the D genome (Schalk et al., 2017). Despite its absence in these wheat types, gluten from all wheats was found to stimulate immune responses in human‐derived T cells in vitro (Suligoj et al., 2013).

Most recently, Pronin et al. (2021) confirmed that the ranges of contents of CD‐active peptides in old and modern wheat cultivars overlap, indicating that the immune‐reactive potential of older bread wheat cultivars was not lower compared to modern cultivars. Furthermore, the content of the 33‐mer peptide remained largely unchanged over time (see Figure 5b). In the light of these observations, it can be concluded that no single wheat type can be recommended as better or safer for reducing or mitigating CD. Environmental growing conditions had an effect on the content of CD‐active peptides in bread wheat (Pronin et al., 2021; Schalk et al., 2017), but genotype has been reported to have a greater effect than environment on the abundance of CD‐active epitopes in durum wheat (Boukid et al., 2017; Prandi et al., 2017). The effects of mineral nutrition have not been determined. The sample set including 60 German winter wheat cultivars were not fertilised but the available soil nitrogen was stated as 50–70 kg/ha, which is much lower than the levels used in conventional high input production systems (up to 200 kg/Ha) while details of nitrogen fertilisation were not provided in other reports (Escarnot et al., 2018; Gélinas & McKinnon, 2016; Hajas et al., 2017; Schopf & Scherf, 2018). This is an important consideration because the availability of nitrogen, and to a lesser extent sulphur, has effects on gluten protein content and composition (Shewry, 2011).

## DO ANCIENT WHEATS INDUCE FEWER INTESTINAL SYMPTOMS?

Several studies have compared traditional types of bread wheat (often referred to as ‘landraces’), which were grown before the introduction of modern plant breeding technologies, but are still grown today on small scale by local farmers in some countries, including Italy, compared with modern types of bread wheat which are grown on a very large scale. Although these older and modern types of durum and bread wheats are genetically similar, small differences in grain composition and in the effects of digestion and gastrointestinal symptoms were reported (Dinu et al., 2018, Spisni et al., 2019). It is likely that at least some of these differences can be explained by the effects of the environment and the agronomic conditions under which the grains were grown and that the same types grown in other regions or with different agronomy would similarly differ in composition. Consequently, there is debate about the significance of the reported differences for effects on health (Shewry, 2018; Shewry et al., 2020). In addition, some differences in inflammatory responses and gastrointestinal symptoms were shown in a number of studies (for reviews see Sofi et al. (2014) and Spisni et al. (2019)). However, many studies were carried out using in vitro or animal systems, which have limited physiological relevance to humans. In addition, the few in vivo human studies that are available have only included small numbers of people (Dinu et al., 2018). The latter does not necessarily invalidate the results but does show that there is a clear need for well‐designed and controlled studies with sufficient statistical power.

Similarly, although it is also clear that ‘landraces’ can be used to increase diversity, nutrient composition and food processing quality in wheat breeding programs (see further below), more robust studies are required to conclude whether this would result in significant impacts on health.

## IS GLUTEN THE CAUSE OF INTOLERANCES IN MANY INDIVIDUALS?

It is estimated that based on confirmed diagnosis, ~1%–7% of the total population may suffer from adverse reactions due to wheat consumption (~1% from CD, ~0.25%–0.5% from wheat allergy [WA]), while variable percentages (1%–6%) have been reported for non‐coeliac wheat sensitivity (NCWS), the values reported being strongly influenced by self‐perception and criteria of evaluation (Capannolo et al., 2015; Sergi et al., 2021). It is an intriguing question why the majority of the total population develops oral tolerance to wheat and other gluten‐containing grains, without any adverse effects. What are the mechanisms leading to symptoms in those who develop CD, WA and NCWS, what differentiates these symptoms from those observed in inflammatory bowel disease and irritable bowel syndrome (IBS) and, what are the mechanisms that protect those who develop oral tolerance to gluten‐containing grains? Unravelling this matter is very complex and challenging (Catassi et al., 2015; Sergi et al., 2021).

The term gluten intolerance is often used by health professionals as a synonym of CD, as well as to indicate that a person experiences improvement of symptoms after starting a gluten‐free diet, while to the general public the term gluten intolerance is also often thought to be the same as gluten allergy. In fact, the term gluten intolerance is not sufficiently specific and it is better to use the specific terms ‘coeliac disease’, ‘wheat allergy’ and ‘non‐coeliac wheat sensitivity’. CD is defined as a chronic small intestinal immune‐mediated gut disease caused by exposure to non‐digested CD‐active peptides which may enter the lamina propria of the small intestine via trans‐cellular or para‐cellular routes, leading to a cascade of reactions causing adaptive immune responses (induced by epitope recognition), inflammation and small intestinal tissue damage, known as coeliac disease, which develops only in genetically predisposed individuals (Lebwohl and Leffler (2015). Some individuals develop allergies to wheat components (wheat allergens) that cause an immediate immune reaction after exposure by either ingestion, or inhalation, or skin contact. Allergic reactions to wheat, in contrast to CD, involve the production of IgE (immunoglobulin) antibodies (for a review see Pasha et al. (2016)).

During recent years a cluster of intestinal and non‐intestinal symptoms (e.g. headache, poor sleep, anxiety, depression) associated with the intake of wheat was attributed to the presence of gluten and described as non‐coeliac gluten sensitivity (NCGS). Avoidance of foods containing gluten was shown to induce a relief of symptoms, which was taken as confirmation that gluten was the causative factor (Biesiekierski et al., 2011). However, follow‐up studies (Ajamian et al., 2021; Biesiekierski et al., 2013) showed that gluten was not the cause and the focus changed to fructans (a class of rapidly fermentable oligo‐ or polysaccharides, consisting of chains of fructose with a terminal glucose molecule) which act as dietary fibre (Fedewa & Rao, 2014) and also form part of the group of components known as FODMAPs (Fermentable Oligo‐, Di‐ and monosaccharides and Polyols). Fructans and other FODMAPs may cause gastrointestinal symptoms, due to gas formation, osmotic fluid fluxes and feelings of bloating of the intestine. These symptoms strongly overlap with symptoms of IBS and reducing FODMAP intake in IBS patients was shown to bring significant relief (Barrett & Gibson, 2012; van Lanen et al., 2021a, 2021b). The observation that FODMAPs rather than gluten in wheat may trigger the ‘sensitivities’ resulted in the term NCGS being changed to NCWS. However, since FODMAPs are clearly related to local intestinal symptoms, it remains challenging to link FODMAPs to the extra‐intestinal symptoms (tiredness, headache, pain in muscles and joints, depression and anxiety) often experienced and other compounds may still play a role (Brouns et al., 2019). In addition to gluten and FODMAPs, wheat contains many other proteins that may play a role in adverse reactions (Huebener et al., 2015; Rej & Sanders, 2019; Sergi et al., 2021). These include enzyme inhibitors (e.g. amylase trypsin inhibitors [ATIs] and serine protease inhibitors [serpins]), low molecular weight gluten‐related proteins (purinins, farinins), globulin storage proteins and wheat germ agglutenins. It is also important to note that isolated wheat gluten, which is often used when testing GF products versus the same products with added gluten, contains significant amounts of non‐gluten proteins, notably ATIs, which can have effects independent of gluten and therefore make it difficult to draw conclusions on the effects of gluten itself (Zevallos et al., 2017).

It is thought that ATIs, together with gluten, may play a role in the initiation of CD and also may cause immune activation and non‐specific allergic intestinal reactions in some individuals who do not suffer from CD (Junker et al., 2012). In a subset of individuals who experienced sensitivity to wheat in the absence of coeliac disease, significantly increased levels of lipopolysaccharide (LPS)‐binding protein (LBP), as well as antibody reactivity to microbial antigens, indicating systemic immune activation were observed (Uhde et al., 2016)^1^.

It was also noted that affected individuals had significantly elevated circulating levels of fatty acid‐binding protein‐2 (a marker of intestinal epithelial damage), which correlated with markers of systemic immune activation. Thus, in the absence of CD, several nonspecific symptoms and factors were present, which overlap with similar symptoms observed in IBS and limit an accurate diagnosis (Catassi et al., 2015; Rej & Sanders, 2019; Sergi et al., 2021). It is therefore important to unravel the roles of individual grain components in triggering symptoms in the reported types of adverse reactions to wheat as well as to develop appropriate diagnostic criteria.

## DIAGNOSIS BY AN EXPERT IS IMPORTANT

The global prevalence of IBS is about 10% (more prevalent in women than in men) but the value may differ depending on the Salerno criteria used and country of study (Oka et al., 2020). The prevalence of individuals who self‐report ‘gluten sensitivity’ is in the range of 6%–13%, but these percentages are unreliable because of the lack of appropriate diagnostic markers and criteria and the real numbers may well be lower (Sergi et al., 2021). Individuals who consider themselves to be sensitive to wheat (gluten) based on self‐diagnosis should consult an appropriate medical setting in which the presence of CD and WA can be excluded and existence of NCWS can be confirmed. The latter can be done on the basis of the disappearance of the symptoms within a pre‐determined period of adhering strictly to a wheat‐free diet, followed by re‐appearance after the re‐introduction of gluten‐containing (wheat) grains in the diet (Catassi et al., 2015). The importance of controlled clinical testing was demonstrated by Capannolo et al. in a study in which 3178 persons suffering from IBS‐like symptoms were admitted by their general practitioners to a gastroenterology unit (Capannolo et al., 2015). Within this large IBS cohort, 392 persons (307 females and 85 males) complained of suffering from symptoms after consuming gluten. During initial screening after admission, it was shown that the percentage of individuals from this selective group suffering from (not previously diagnosed) CD was 6.63%, which is much higher than the general population prevalence of about 1%. In addition, the positive predictive value of the gluten‐related symptoms, defined as the probability that someone with symptoms related to gluten really suffers from symptoms after consuming gluten/wheat was only 7% (Capannolo et al., 2015). To put the latter in a correct prevalence perspective, 7% of an IBS cohort (which usually represents 10%–12% of total population) would equal a NCWS prevalence of close to 1% of the total population.

**FIGURE 6:**
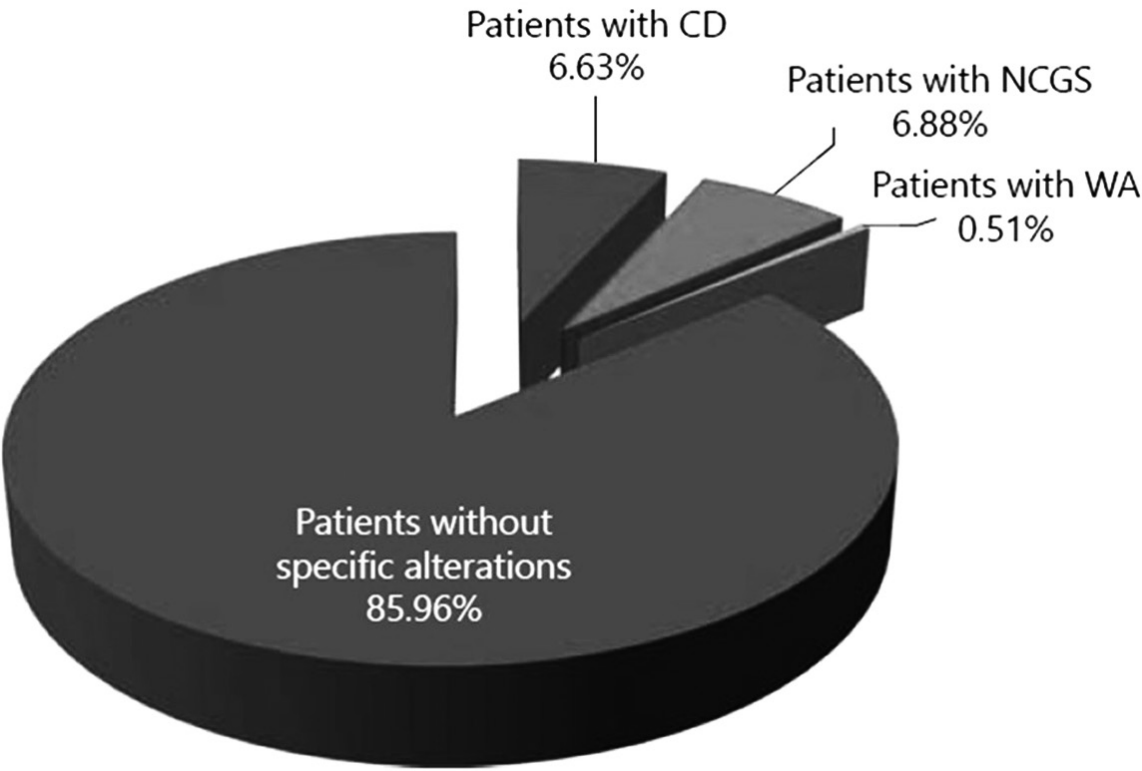
From an irritable bowel syndrome sub‐cohort, 392 persons (12.3% of the cohort, 307 females and 85 males), complained of gastrointestinal symptoms after the consumption of gluten‐containing foods. Of these patients 6.63% were diagnosed to suffer from coeliac disease and 0.51% from wheat allergy (WA). The remaining 337 persons were all put on a 6‐months strictly gluten‐free (GF) diet, followed by a reintroduction of gluten‐containing grains in their diet for 1 month. Despite their self‐diagnosis to be gluten sensitive, 85.96% of these individuals showed no specific reaction to gluten. CD, coeliac disease; NCGS, non‐coeliac gluten sensitivity. With permission from Capannolo et al. (2015)

From the above it is clear that appropriate diagnosis is an essential first step to diagnose the presence or absence of CD and WA and to justify a major change in lifestyle, with a life‐long gluten‐free diet, excluding all gluten‐containing cereals with risks of developing nutritional deficiencies unless appropriately managed. In addition, undiagnosed CD increases the risk of other associated diseases. (See Figure 6 and related explanation).

## CONCLUDING REMARKS

It is clearly not correct to apply the term ‘ancient’ to older types of bread and durum wheats, which may correspond to land races or the early products of plant breeding. These rarely date back more than a century and may be more correctly termed ‘heritage’ or ‘heirloom’ wheats. The term is more widely used for einkorn, emmer, spelt and Khorasan wheats. Although the application of the term to spelt can be criticised, it nevertheless provides a working definition to discriminate these types from modern bread and durum wheats. Comparisons of older (heritage) and modern types of bread wheat grown and analysed under identical conditions have shown that older types contain more protein, including total gluten and gliadin (which is the major source of CD‐active gluten peptides). Einkorn, emmer and durum do not contain the D genome encoding the α‐gliadin which is digested to release the ‘33‐mer’ peptide (α2‐gliadin p56–88) and is a strong CD trigger in genetically susceptible individuals. However, they do contain other CD‐active peptides and their contents clearly overlap between ancient and modern wheat cultivars. It can therefore be concluded that no single wheat type can be recommended as better or safer for reducing or mitigating CD. Although older and modern types of durum and bread wheats are genetically similar, small differences in the compositions of grain samples occur, some of which may result from differences in the environment and agronomic conditions under which the grains are grown. However, the relevance of these to impacts on health has not been established. Although most of the literature on CD and WA focuses on the role of gluten proteins it should be noted that other wheat proteins may trigger allergic, immune and inflammatory responses in susceptible individuals, including the ATIs. The initial suggestion that gluten was the prime cause of self‐reported wheat sensitivity has been disproved in more recent studies which have shown a role of fermentation of FODMAPs resulting in gas formation and bloating. Despite this observation, FODMAPs cannot explain inflammatory and immune reactions observed in some individuals, which indicate that other components are inducing factors. Finally, the impact of gluten‐free diets on health and quality of life warrants that accurate medical diagnosis is essential before deciding to exclude gluten‐containing grains from diets, especially because strict adherence to GF foods is challenging, costly and may be socially isolating. In addition, GF foods are often of less favourable nutritional composition, have been linked to poor micronutrient and fibre intakes, which was shown to reduce the bacterial richness and microbiota composition in non‐celiac individuals in an unfavourable way, and require dietetic guidance to help avoid undesired effects on health outcomes (Caio et al., 2020; Jansson‐Knodell & Rubio‐Tapia, 2021; Johnston et al., 2017; Lerner et al., 2019; Littlejohns et al., 2021). The development of appropriate diagnostic tools and criteria and a clear diagnosis is therefore a priority.

## CONFLICTS OF INTEREST

The authors report no conflicts of interest to declare that are relevant to the content of this article.

## AUTHOR CONTRIUTIONS

All authors contributed to the preparation of this manuscript and have read and approved the final text.

## Data Availability

Data sharing not applicable – no new data generated, or the article describes entirely theoretical research.

## Footnotes

1 LPS (also called endotoxins) are glycolipids found in the outer membrane of gram‐negative bacteria that bind to immune modulating receptors (CD14‐TLR4/MD2) and promote the secretion of pro‐inflammatory cytokines.
